# Supplementary material for: Associate toxin-antitoxin with CRISPR-Cas to kill multidrug-resistant pathogens
Source: Nat Commun. 2023 Apr 12;14:2078. doi: 10.1038/s41467-023-37789-y (PMC10097628; doi:10.1038/s41467-023-37789-y)
Supplement: Supplementary file 1 — Supplementary information [file 41467_2023_37789_MOESM1_ESM.pdf]

Supplemental information for

Associate Toxin-Antitoxin

with CRISPR-Cas to

Kill Multidrug-Resistant Pathogens

Rui Wang, Xian Shu, Huiwei Zhao, Qiong Xue, Chao Liu, Aici Wu, Feiyue Cheng, Lingyun Wang, Yihan Zhang, Jie Feng, Nannan Wu, Ming Li

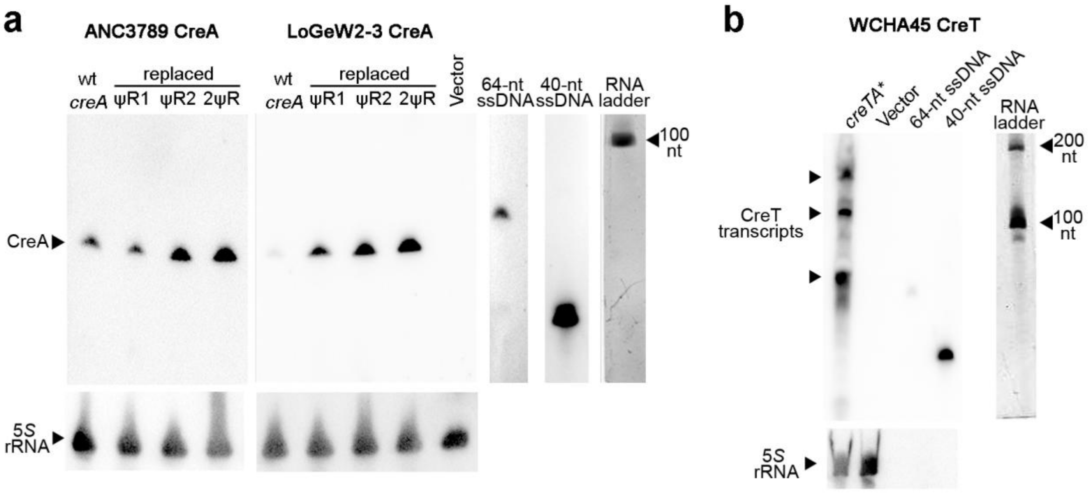

Supplementary Fig. 1. Northern blotting of the CreA (a) or CreT (b) RNA of *Acinetobacter* species. a. *A. baumannii* AYE cells transformed by plasmids carrying the wild-type (wt) or repeat-replaced *creA* gene (from *Acinetobacter* sp. ANC3789 or LoGeW2-3) were subjected to total RNA extraction and blotting analysis. For repeat replacement, the ΨR1 or ΨR2 element, or both (2ΨR), were replaced by the CRISPR repeat of AYE. All data were acquired from at least three independent replicates with similar results. **b.** *A. baumannii* AYE cells transformed by plasmids carrying the ΨR2-replaced WCHA45 *creTA* operon was similarly subjected to Northern blotting. Cells containing the empty vector served as the negative control. 5*S* rRNA was proved as the internal control. A 100-nt RNA ladder and two biotin-labeled oligonucleotides (of 64-nt and 40-nt, respectively) were co-electrophoresed with the RNA samples. All data were acquired from at least three independent replicates with similar results. Source data are provided as a Source Data file.

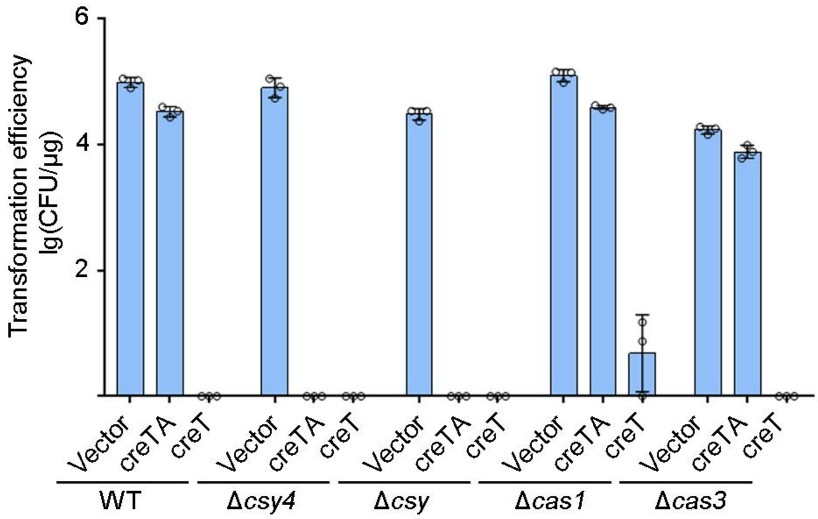

Supplementary Fig. 2. The antitoxic function of WCHA45 CreA relies on the Csy complex, not on Cas1 or Cas3. AYE cells were transformed by plasmids carrying only the *creT* gene or those carrying both *creT* and *creA* genes. Note that, for this assay, the ΨR2-replaced (by the CRISPR repeat of AYE) version of WCHA45 *creA* was used. When not repressed, the toxicity of *creT* caused a 5-log reduction. The transformation efficiency is shown as mean value ± s.d. (n=3 biological replicates). Source data are provided as a Source Data file.

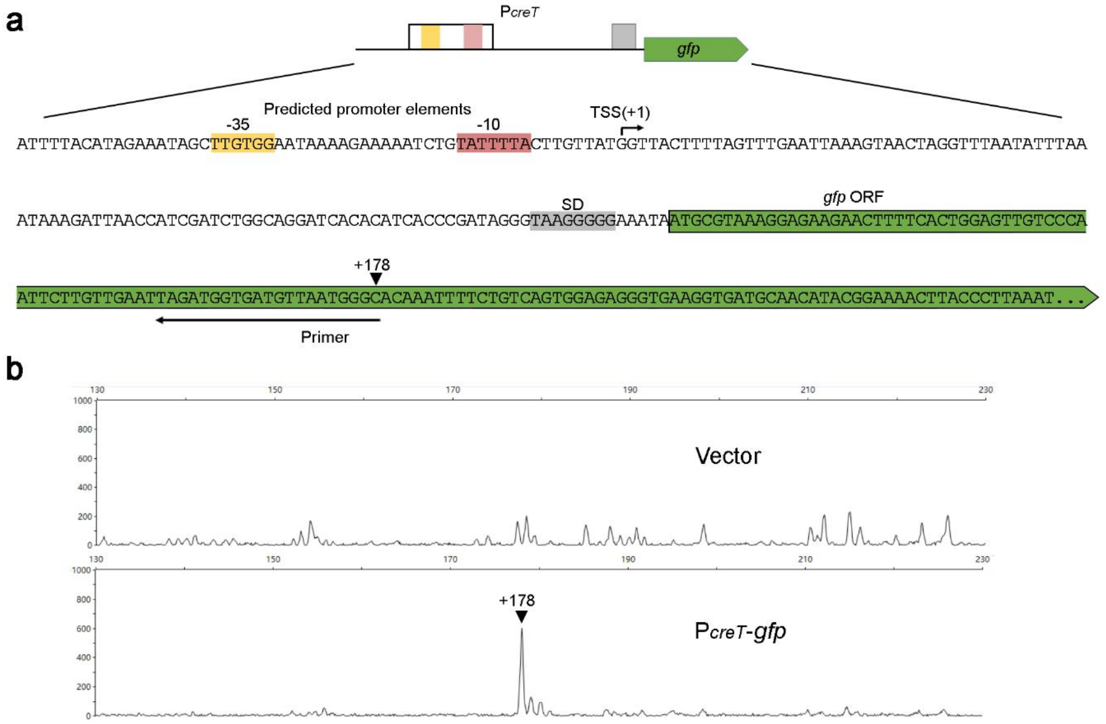

Supplementary Fig. 3. Primer extension assay to determine the transcription start site (TSS) of *creT*. a. Scheme showing the experimental design. Plasmid carrying the P_{creT}-*gfp* construct was introduced into AYE cells, and the total RNA were used for primer extension. The primer was designed against the *gfp* RNA transcript and 5'-labeled by FAM (see the Methods part). The DNA products of primer extension assay was subjected to fragment analysis. **b.** The results of primer extension. TSS was identified according to the fragment size of the DNA products. Cells containing the empty vector were used as the negative control.

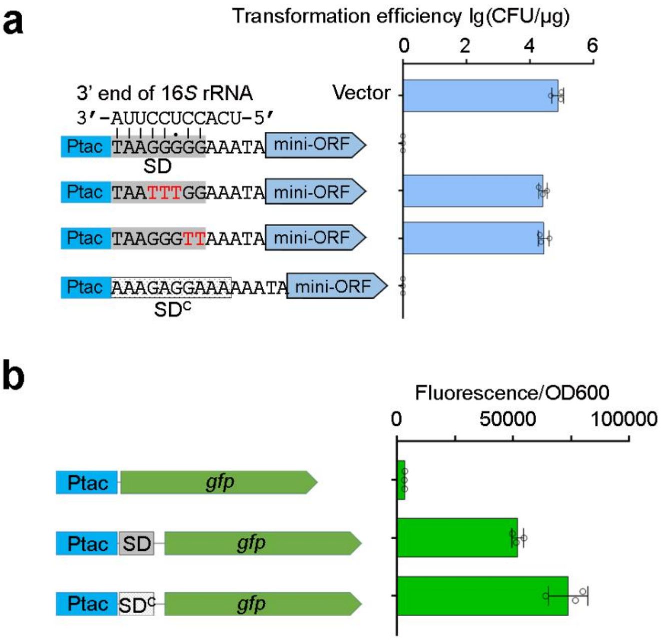

Supplementary Fig. 4. The predicted SD motif of *creT* is critical for its toxicity. a. Transformation of AYE cells by plasmids carrying a wt, SD-mutated, or SD-replaced *creT* gene. Mutated nucleotides are shown in red. SD^c, a canonical *E. coli* SD sequence used to improve protein synthesis (ref. [27]). The SD and mini-ORF of WCHA45 *creT* gene were put under the control of a *tac* promoter (P_{tac}). The base pairing potential of SD motif to the 3' end of 16*S* rRNA is depicted. Data are presented as mean value ± s.d. (n=3 biological replicates). **b.** Functional validation of the SD motif predicted for WCHA45 *creT*. AYE cells expressing a *gfp* gene was subjected to simultaneous OD600 and fluorescence measurements. The *gfp* gene was under the control of P_{tac} with the predicted SD motif of *creT* or a canonical SD motif (SD^c) (ref. [27]). A SD-minus construct was used as the negative control. Data are presented as mean value±s.d. (n=3 biological replicates). Source data are provided as a Source Data file.

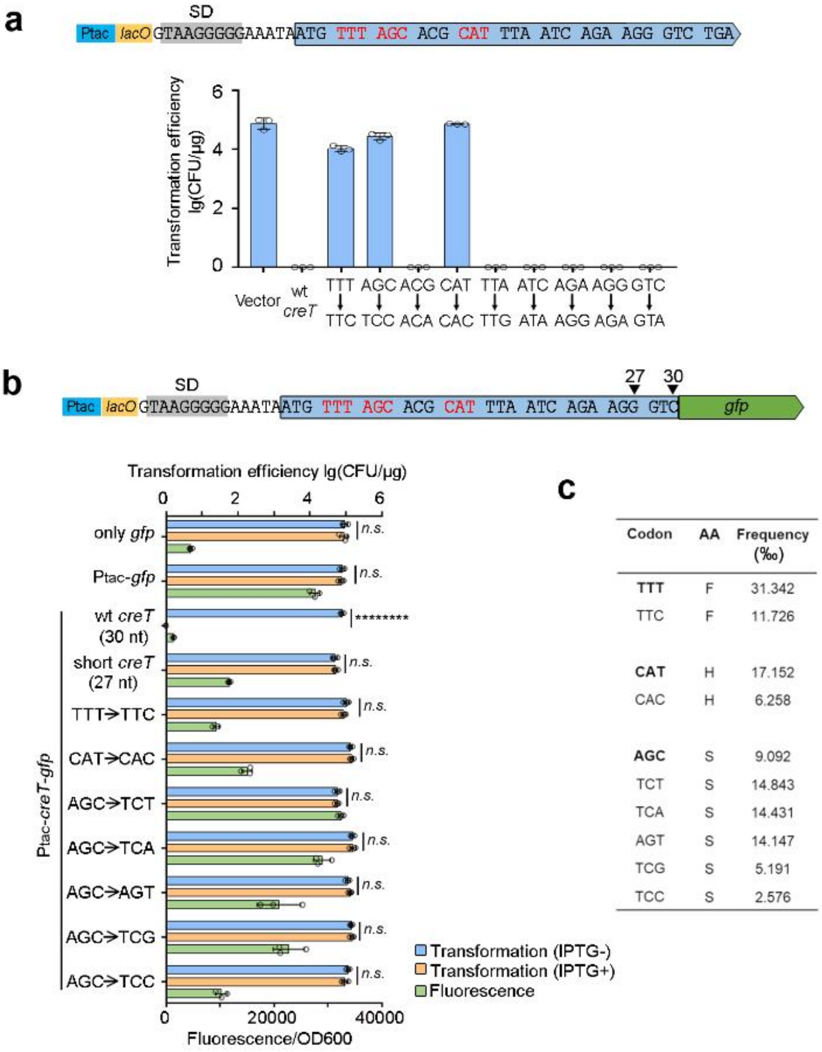

Supplementary Fig. 5. Toxicity of CreT did not rely on its potential protein product. a. Synonymous mutation analysis of *creT*. The SD and mini-ORF of *creT* was designed under the control of P_{tac} and the *lacO* operator. Toxicity was lost when the red codons were mutated. The transformation efficiency is shown as mean value ± s.d. (n=3 biological replicates). **b.** Fusion of *creT* and *gfp*, and the transformation of AYE cells by plasmids carrying each *creT-gfp* fusion. The wide-type (wt) *creT* gene was first fused with *gfp* and then the 2nd, 3rd, and 5th codons (indicated in red) were separately replaced by their synonymous codons. A shortened *creT* (to 27 nt) was also fused to *gfp* to provide a control. The efficiency was determined by plating on inducing (IPTG+) or non-inducing (IPTG-) medium. Individual colonies on the non-inducing medium were inoculated into liquid medium and subjected to fluorescence measurement after cultured to the log phase. Plasmids carrying a promoter-lacking *gfp* gene (only *gfp*) or a P_{tac}-controlled *gfp* gene (P_{tac}-*gfp*) were used as controls. Data are presented as mean value ± s.d. (n=3 biological replicates). Two-tailed Student's *t* test [*n.s.*, not significant (P > 0.05); ********P < 0.00000001. P=0.697635460, 0.642430855, 4.51548E-09, 0.60262183, 0.269517139, 0.476578403, 0.764254414, 0.796767397, 0.188402074, 0.188402074, 0.642005051, 0.215543339 (top to bottom)]. **c.** The usage frequency (among all codons) in *A. baumannii* AYE is given for each phenylalanine (F), histidine (H), or serine (S) codon. Source data are provided as a Source Data file.

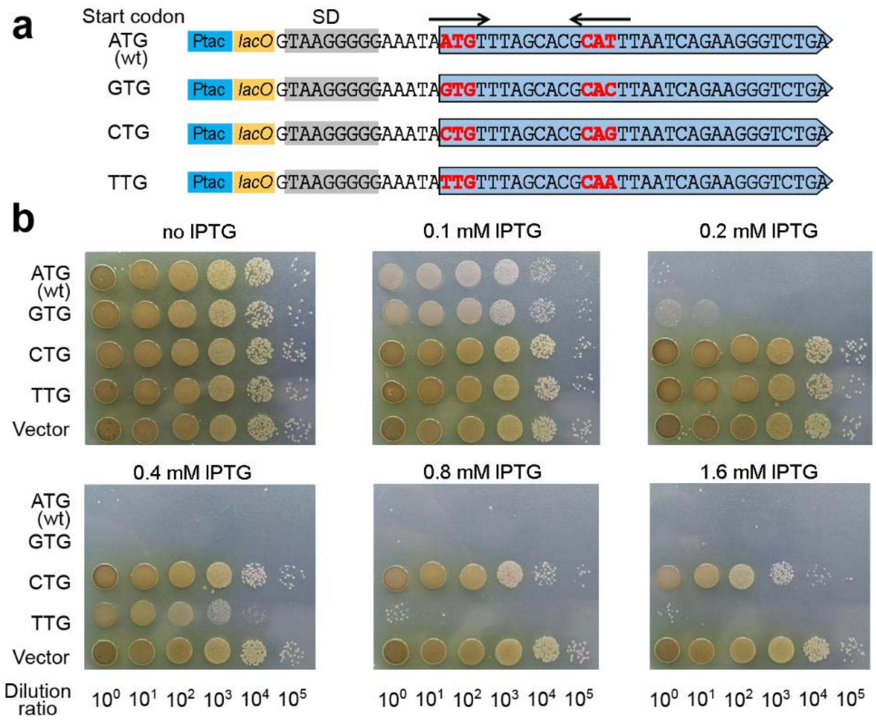

Supplementary Fig. 6. Toxicity of CreT corelates with the efficiency of its start codon. a. Mutation of the start codon AUG and its complementary nucleotides (in red). The inverted repeats underlying the hairpin structure of CreT RNA are indicated by a pair of black arrows. Note that these two repeats were simultaneously mutated to keep their complementarity. The wt (ATG) or mutated (GTG, CTG, and TTG) *creT* gene was under the control of P_{tac} and a *lacO* operator. **b.** Growth of *A. baumannii* AYE cells expressing a wt or mutated *creT* on plates containing different amounts of IPTG. The plasmid used to express *creT* also carried a *lacI* gene. Liquid cultures of AYE were serially diluted before spotted on plates. The experiments were repeated independently at least 3 times and the representative results were shown.

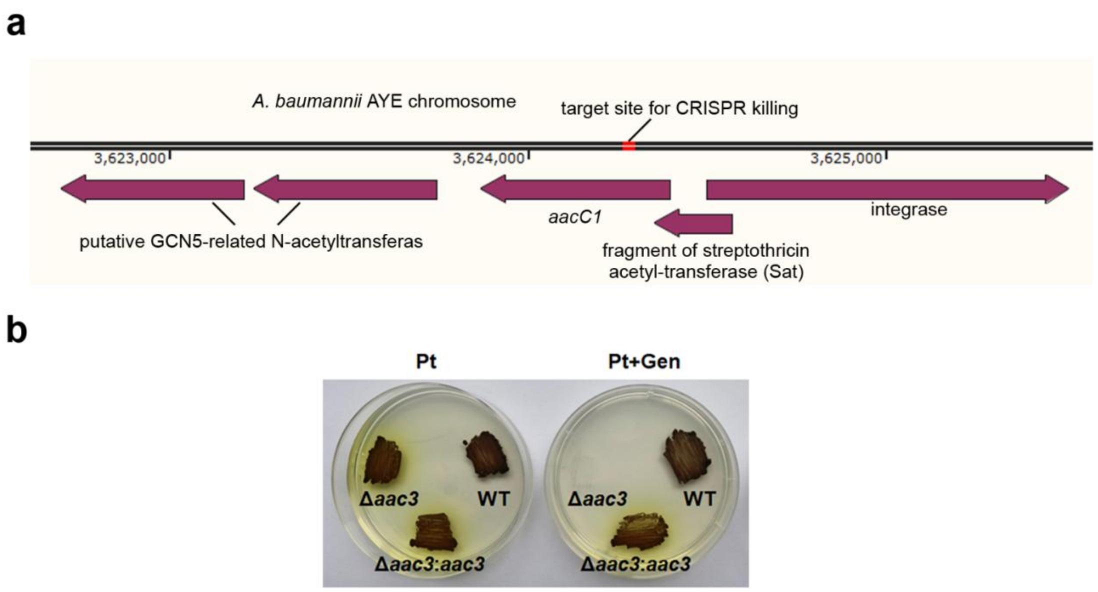

Supplementary Fig. 7. The *aac3* (annotated *aacC1* in the NCBI GenBank) gene decides the gentamycin resistance of *A. baumannii* AYE. a. Genomic context of *aac3*. The target site selected for designing CRISPR antimicrobials is indicated. **b.** Growth of AYE cells on plates containing only potassium tellurite (Pt), or Pt plus gentamycin (Gen). The *aac3* gene was in-frame deleted to generate the Δ*aac3* mutant. WT and Δ*aac3* AYE cells were transformed by an empty vector (conferring Pt resistance), and the Δ*aac3:aac3* cells contained a vector-born *aac3*.

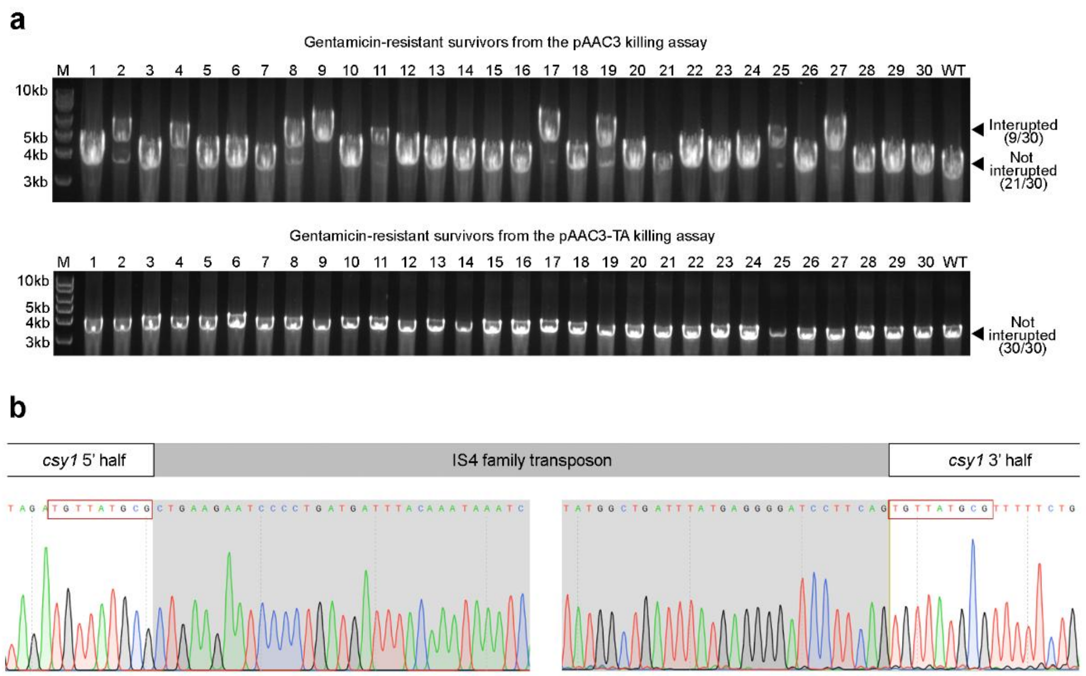

Supplementary Fig. 8. The *csy* operon was frequently interrupted by an IS element in AYE cells surviving from the pAAC3 killing assay, but not in those surviving from the pAAC3-TA killing assay. a. Surviving colonies on gentamycin-containing plates were randomly selected and subjected to colony PCR. The ~3.8 kb *csy* operon (consisting of *csy1*, *csy2*, *csy3*, and *csy4*) was amplified using specific primers. The ratio of cells containing an interrupted or not interrupted *csy* operon is given besides the gel. The experiments were repeated independently for at least 3 times and the representative results were shown. **b.** An example sequencing result illustrating the interruption of *csy1* by the IS element (IS4 family, genomic position: 1271633-1272960). The duplicated target sequence is labeled with a red box.

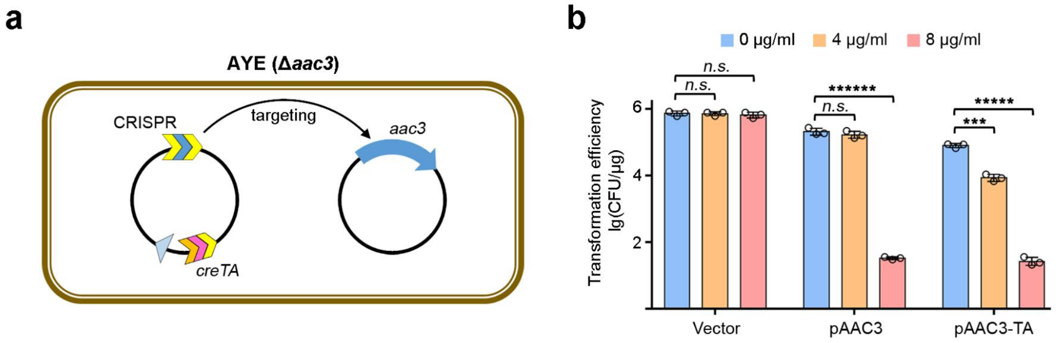

Supplementary Fig. 9. CRISPR-Cas cured a plasmid-born AR gene more completely and efficiently in the presence of CreTA. a. Schematic depicting the experimental design of the plasmid curing assay. A plasmid carrying the *aac3* gene was pre-introduced into the Δ*aac3* cells of *A. baumanni* AYE, and these resulting cells were then transformed by a second plasmid (pAAC3-TA) carrying the mini-CRISPR with an *aac3*-targeting spacer and the *creTA* operon. **b.** Transformation efficiency of the empty vector, pAAC3 (lacking the *creTA* module), and pAAC3-TA on medium containing potassium tellurite (30 μg/ml, selecting for the second plasmid) and 0, 4, or 8 μg/ml gentamycin. Data are presented as mean value ± s.d. (n=3 biological replicates). Two-tailed Student's *t* test [*n.s.*, not significant ($P > 0.05$); ***$P < 0.001$; *****$P < 0.00001$; ******$P < 0.000001$; P=0.904823005, 0.485270998, 0.335071539, 4.73E-07, 0.000168199, 1.67E-06 (left to right)]. Source data are provided as a Source Data file.

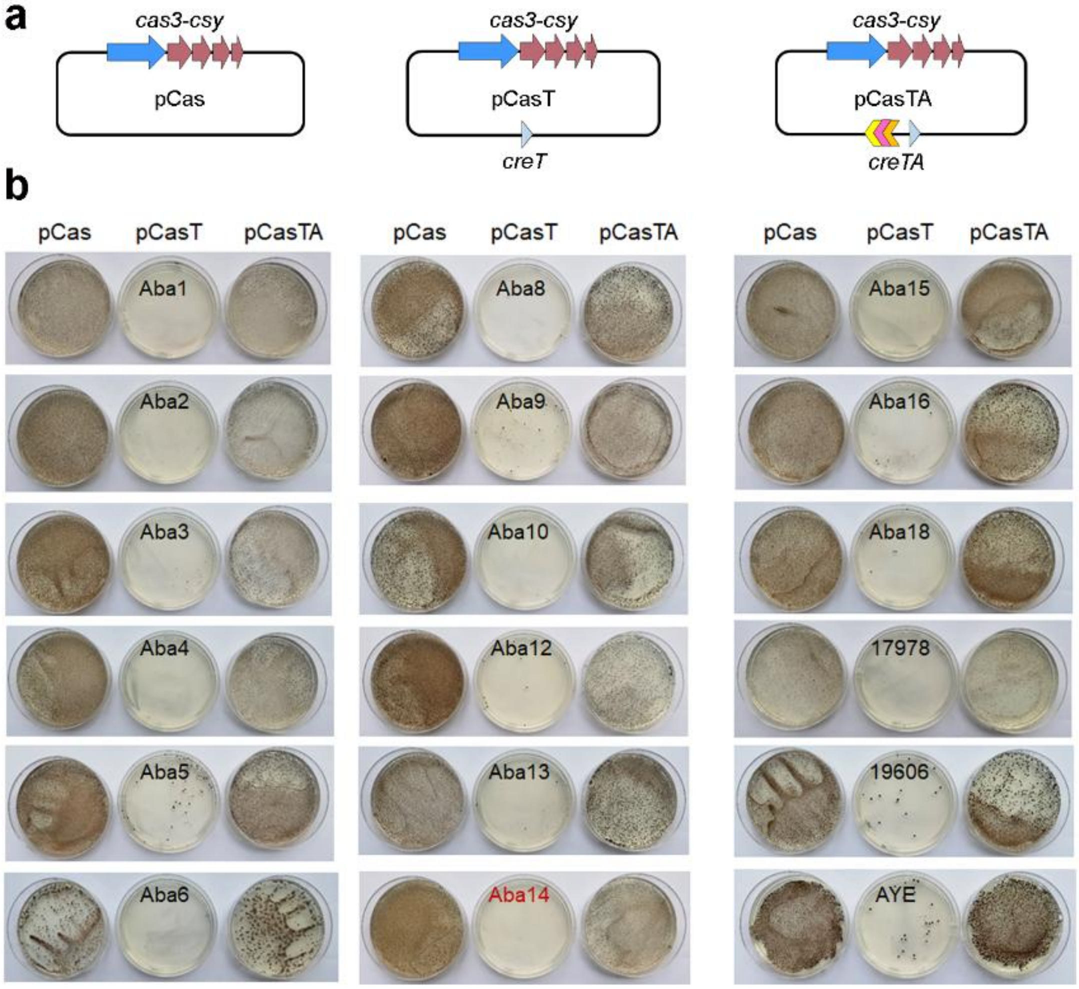

Supplementary Fig. 10. WCHA45 CreTA and AYE CRISPR-Cas are active and compatible in all of 18 clinical isolates (numbered from Aba1 to Aba18) and two type strains (ATCC 17978 and 19606) of *A. baumannii*. a. Schematic depiction of plasmid design. pCas carries the *cas3-csy* operon from *A. baumannii* AYE, pCasT also carries the *creT* gene of *Acinetobacter* sp. WCHA45, and pCasTA further expresses its *creA* gene (with ΨR2 replaced by the CRISPR repeat of AYE). **b.** Example photos showing the colonies formed on selective plates (containing Pt) when *A. baumannii* cells (of different isolates/strains) were transformed by these plasmids via conjugation. The clinical isolate Aba14 (in red) was selected for the CRISPR antimicrobial assay in Fig. 6. See Supplementary Data 1 for the MDR feature of clinical isolates.

Source data for Supplementary Figures

Source data for Supplementary Fig. 1a

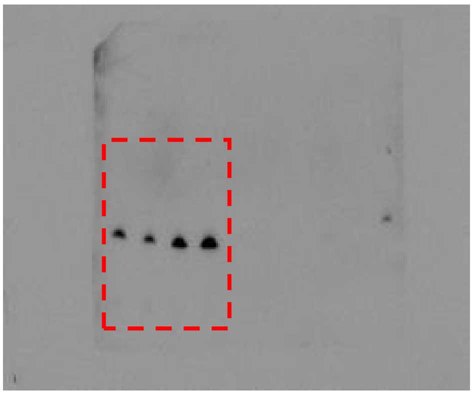

ANC3789 CreA probe

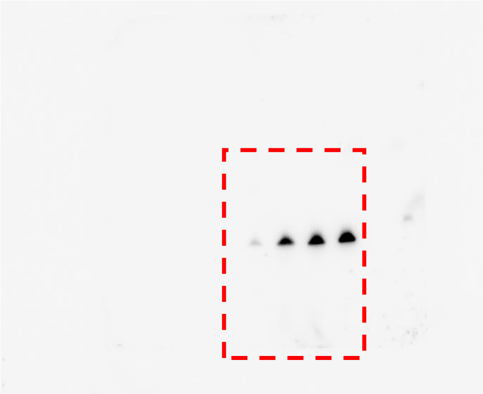

LoGeW2-3 CreA probe

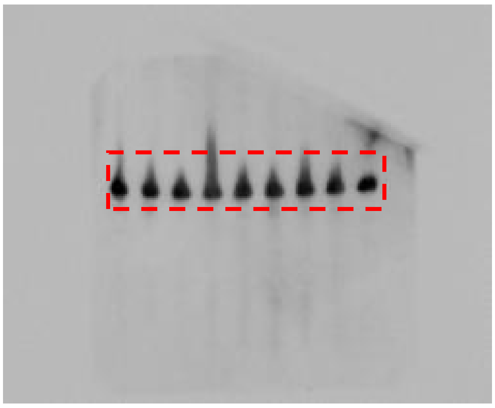

5S rRNA probe

ladder

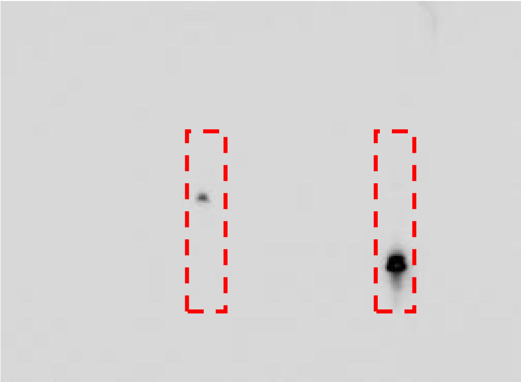

ssDNA-biotin marker

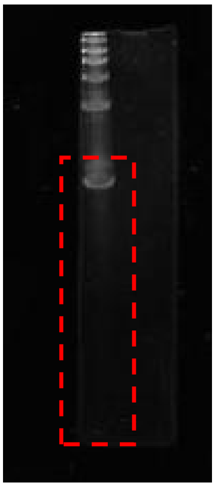

RNA

Source data for Supplementary Fig. 1b

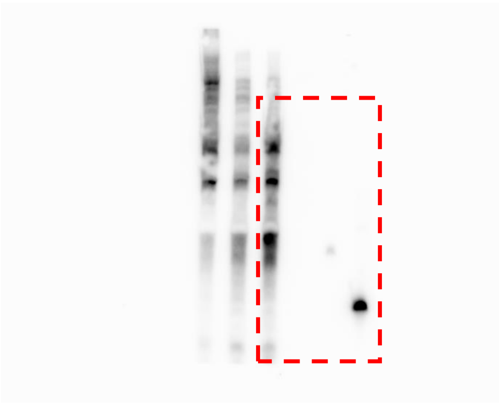

CreT probe

ladder

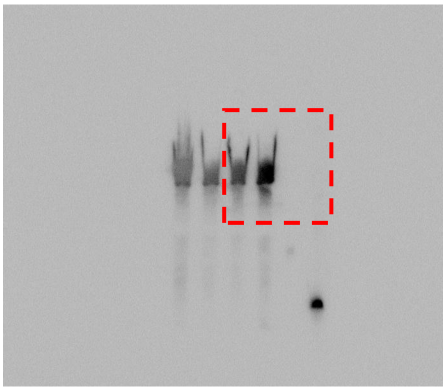

5S rRNA probe

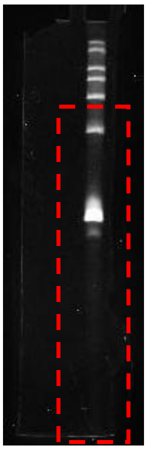

RNA

Source data for Supplementary Fig. 8a

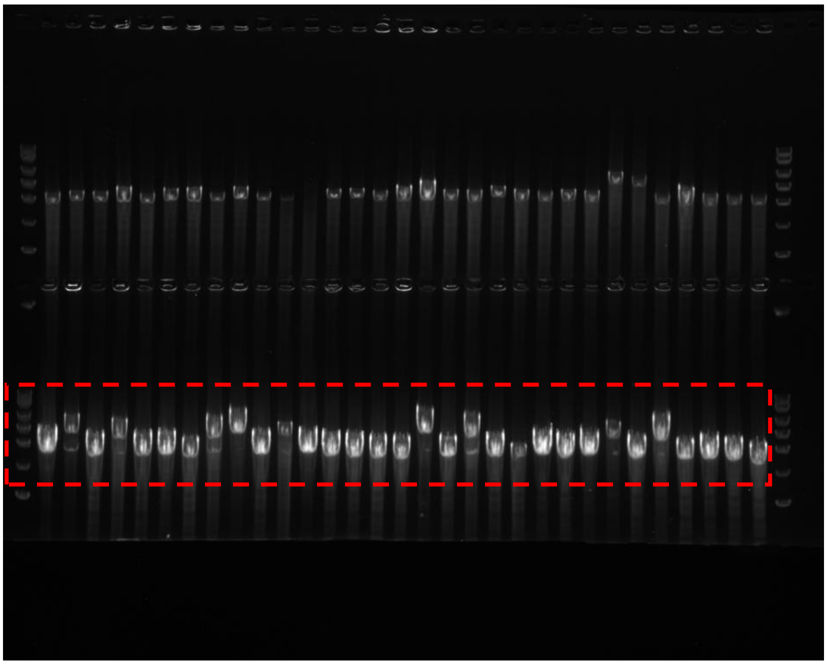

Amplification of the *csy* operon of gentamicin-resistant survivors from the pAAC3 killing assay.

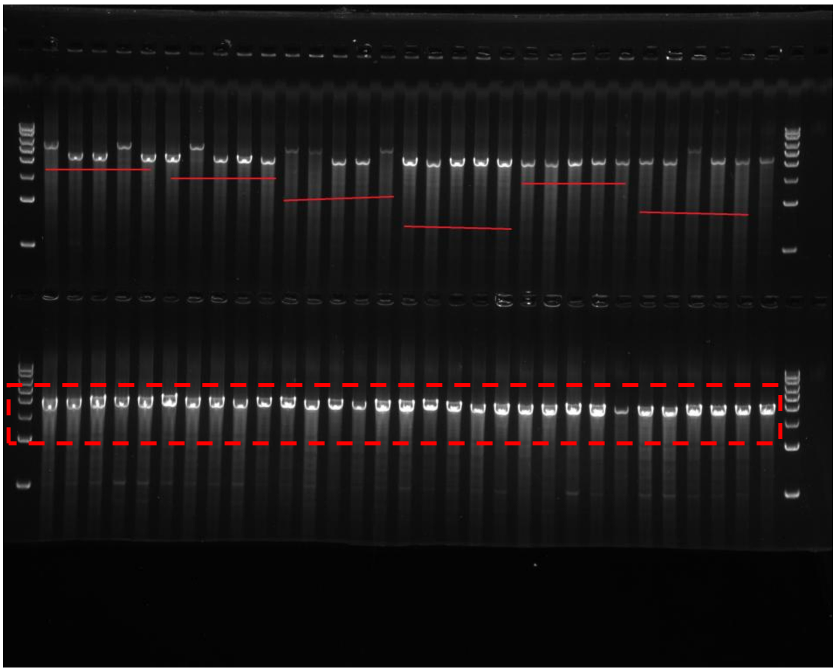

Amplification of the *csy* operon of gentamicin-resistant survivors from the pAAC3-TA killing assay.
