# Supplementary material for: Associate toxin-antitoxin with CRISPR-Cas to kill multidrug-resistant pathogens
Source: Nat Commun. 2023 Apr 12;14:2078. doi: 10.1038/s41467-023-37789-y (PMC10097628; doi:10.1038/s41467-023-37789-y)
Supplement: Supplementary file 2 — Reporting Summary [file 41467_2023_37789_MOESM2_ESM.pdf]

nature portfolio

Corresponding author(s): Ming Li

Last updated by author(s): Mar 30, 2023

Reporting Summary

Nature Portfolio wishes to improve the reproducibility of the work that we publish. This form provides structure for consistency and transparency in reporting. For further information on Nature Portfolio policies, see our Editorial Policies and the Editorial Policy Checklist.

Statistics

For all statistical analyses, confirm that the following items are present in the figure legend, table legend, main text, or Methods section.

n/a	Confirmed	
☐	☒	The exact sample size (*n*) for each experimental group/condition, given as a discrete number and unit of measurement
☐	☒	A statement on whether measurements were taken from distinct samples or whether the same sample was measured repeatedly
☐	☒	The statistical test(s) used AND whether they are one- or two-sided<br>*Only common tests should be described solely by name; describe more complex techniques in the Methods section.*
☒	☐	A description of all covariates tested
☒	☐	A description of any assumptions or corrections, such as tests of normality and adjustment for multiple comparisons
☐	☒	A full description of the statistical parameters including central tendency (e.g. means) or other basic estimates (e.g. regression coefficient) AND variation (e.g. standard deviation) or associated estimates of uncertainty (e.g. confidence intervals)
☐	☒	For null hypothesis testing, the test statistic (e.g. *F*, *t*, *r*) with confidence intervals, effect sizes, degrees of freedom and *P* value noted<br>*Give P values as exact values whenever suitable.*
☒	☐	For Bayesian analysis, information on the choice of priors and Markov chain Monte Carlo settings
☒	☐	For hierarchical and complex designs, identification of the appropriate level for tests and full reporting of outcomes
☒	☐	Estimates of effect sizes (e.g. Cohen's *d*, Pearson's *r*), indicating how they were calculated

Our web collection on statistics for biologists contains articles on many of the points above.

Software and code

Policy information about availability of computer code

Data collection	No software was used for data collection.
Data analysis	A published script (https://doi.org/10.5281/zenodo.4586956) was used to analyze the RNA-seq data. Sequence alignments were constructed and viewed using the GeneDoc software (version 2.6.002). RNA secondary structure was predicted using the RNAfold webserver. Putative RNA interactions were predicted using the IntaRNA webserver (Freiburg RNA Tools). Promoter elements were predicted using the BPROM program (Softberry tool). Microsoft Excel was used to analyze the data, and GraphPad Prism (version 7.00) was used to generate the plots. The graphs were then modified in Adobe Illustrator (version 24.0) to construct the final figures.

For manuscripts utilizing custom algorithms or software that are central to the research but not yet described in published literature, software must be made available to editors and reviewers. We strongly encourage code deposition in a community repository (e.g. GitHub). See the Nature Portfolio guidelines for submitting code & software for further information.

Data

Policy information about availability of data

All manuscripts must include a data availability statement. This statement should provide the following information, where applicable:

- Accession codes, unique identifiers, or web links for publicly available datasets
- A description of any restrictions on data availability
- For clinical datasets or third party data, please ensure that the statement adheres to our policy

All relevant data are included in the paper and/or its supplementary information files. The raw data for the RNA-seq experiments in Fig. 2 were deposited to the

National Center for Biotechnology Information (NCBI) with the BioProject accession number PRJNA950041 (https://www.ncbi.nlm.nih.gov/sra/PRJNA950041). All strains and plasmids are available from the corresponding author upon request; requests will be answered within 2 weeks.

Human research participants

Policy information about studies involving human research participants and Sex and Gender in Research.

Reporting on sex and gender	N/A.
Population characteristics	N/A.
Recruitment	N/A.
Ethics oversight	N/A.

Note that full information on the approval of the study protocol must also be provided in the manuscript.

Field-specific reporting

Please select the one below that is the best fit for your research. If you are not sure, read the appropriate sections before making your selection.

- [x] Life sciences
- [ ] Behavioural & social sciences
- [ ] Ecological, evolutionary & environmental sciences

For a reference copy of the document with all sections, see nature.com/documents/nr-reporting-summary-flat.pdf

Life sciences study design

All studies must disclose on these points even when the disclosure is negative.

Sample size	The electro-transformation assays were performed in triplicate, while the conjugation assays performed in six replicates. For each experimental setting of the fluorescence and qPCR assays, three independent biological replicates were included. And for the qPCR assay, each biological replicate was examined in triplicate. No statistical methods were used to predetermine the sample size. The regular sample size of 3 (or 6) was selected, which is sufficient to avoid random variations.
Data exclusions	No data was excluded.
Replication	All transformation assays were replicated for at least two times. Northern blotting and dilution plating assays were replicated for three times for confirmation. Similar results were obtained during these replications. See 'Sample size' for the information of biological/technical replicates in different assays.
Randomization	Bacterial colonies were randomly selected for biological replicates.
Blinding	Blinding was not relevant to this study, since no manual counting or scoring was performed to obtain data. Group allocation was not relevant to this study (e.g., the cell culture from the same tube was used for transformation by different plasmids, and the plasmid from the same tube was used to transform different strains).

Reporting for specific materials, systems and methods

We require information from authors about some types of materials, experimental systems and methods used in many studies. Here, indicate whether each material, system or method listed is relevant to your study. If you are not sure if a list item applies to your research, read the appropriate section before selecting a response.

Materials & experimental systems

n/a	Involved in the study	
x		Antibodies
x		Eukaryotic cell lines
x		Palaeontology and archaeology
x		Animals and other organisms
x		Clinical data
x		Dual use research of concern

Methods

n/a	Involved in the study	
x		ChIP-seq
x		Flow cytometry
x		MRI-based neuroimaging
