# Supplementary material for: Associate toxin-antitoxin with CRISPR-Cas to kill multidrug-resistant pathogens
Source: Nat Commun. 2023 Apr 12;14:2078. doi: 10.1038/s41467-023-37789-y (PMC10097628; doi:10.1038/s41467-023-37789-y)
Supplement: Supplementary file 6 — Description of Additional Supplementary Files [file 41467_2023_37789_MOESM6_ESM.pdf]

Title: Supplementary Data 1:
Description: The MDR profile of clinical A. *baumannii* isolates used in this study.

Title: Supplementary Data 2:
Description: WCHA45 creTA and its homologs.

Title: Supplementary Data 3:
Description: Strains, plasmids, and oligonucleotides used in this study
